# The use of technology in the treatment of youth with eating disorders: A scoping review

**DOI:** 10.1186/s40337-022-00697-5

**Published:** 2022-11-24

**Authors:** Rachel Dufour, Kaylee Novack, Louis Picard, Nicholas Chadi, Linda Booij

**Affiliations:** 1 Sainte-Justine Hospital Research Centre, Montreal, Canada; 2 Department of Psychology, Concordia University, Montreal, Canada; 3 Department of Psychiatry and Addictology, Université de Montréal, Montreal, Canada; 4 Division of Adolescent Medicine, Department of Pediatrics, Université de Montréal, Montreal, Canada; 5 Department of Psychiatry, McGill University, Montreal, Canada; 6 Division of Adolescent Medicine, Department of Pediatrics, Sainte-Justine University Hospital Centre, 3175 Chemin de La Côte-Ste-Catherine, Montreal, QC H3T 1C5 Canada; 7 Faculty of Medicine, Université de Montréal, Montreal, Canada

**Keywords:** Eating disorder, Anorexia nervosa, Bulimia nervosa, Youth, Adolescent, Young adult, Technology-based, Telehealth, Telemedicine, Virtual therapy

## Abstract

**Background:**

Adolescence and young adulthood is a high-risk period for the development of eating disorders. In recent years, there has been an increase in use of technology-based interventions (TBIs) for the treatment of eating disorders. The objective of this study was to determine the types of technology used for eating disorder treatment in youth and their effectiveness.

**Methods:**

A scoping review was conducted according to PRISMA-ScR guidelines. Four databases were searched. Eligible articles included: (1) a TBI (2) participants with a mean age between 10- and 25-years and meeting DSM-IV or DSM-5 criteria for any eating disorder and (3) qualitative or quantitative designs. Quantitative and qualitative studies were assessed for quality.

**Results:**

The search identified 1621 articles. After screening of titles and abstracts, 130 articles were read in full and assessed for eligibility by two raters. Forty-nine (29 quantitative and 20 qualitative, observational, or mixed methods studies) met inclusion criteria. Quality ratings indicated that 78% of quantitative studies had a low risk of bias and 22% had a moderate risk. Technologies reviewed in our study included videoconference therapy, mobile applications, and online self-help. We considered interventions used both within sessions with clinicians as well as those used in between sessions by patients alone. Fifteen of 18 (83%) quantitative studies found that TBIs reduce eating disorder symptomatology, with nine of those reporting medium-to-large effect sizes. Qualitative data was of high quality and suggested that virtual interventions are acceptable in this population.

**Conclusions:**

Although identified studies are of high quality, they are limited in number. More research is needed, particularly regarding videoconferencing and mobile applications. Nonetheless, TBIs show promise for the treatment of eating disorders in youth.

*Trial registration*: Not applicable.

**Supplementary Information:**

The online version contains supplementary material available at 10.1186/s40337-022-00697-5.

## Plain English summary

The use of virtual care to treat adolescents and young adults with eating disorders has increased in recent years. To better understand what types of technologies are being used in virtual care and these technologies’ effectiveness, we systematically reviewed research studies that discussed the use of any technology to treat youth (which was defined as young people aged 25 years or under) with an eating disorder. We found 49 relevant studies. They revealed that videoconference therapy, mobile applications, and online self-help are most frequently used for virtual treatment. Of the 18 studies evaluating the effectiveness of these technologies, 15 found virtual care to be effective for reducing eating disorder symptoms. Studies describing patient experiences with these technologies, of which there were nine, suggested that patients overall were satisfied with receiving virtual care. Although these results are promising, research on virtual treatment is still lacking, especially on videoconferencing therapy and mobile applications.

**Supplementary Information:**

The online version contains supplementary material available at 10.1186/s40337-022-00697-5.

## Background

Eating disorders (EDs) affect individuals of all ages, but adolescents are particularly at risk of developing EDs during this critical period of their development [1]. Evidence-based treatments such as family-based treatment (FBT) and cognitive behavioral therapy (CBT) are effective in reducing ED symptoms and promoting recovery in youth with EDs [2, 3]. However, it has been suggested that many children, adolescents, and young adults (hereafter referred to as “youth”) meeting criteria for an ED may not receive these effective therapies for a number of reasons, including limited treatment seeking behaviour [4–6], limited access to specialized care, and stigma surrounding the illness and treatments, among others [7]. More specifically, lack of provider training as well as limited capacity to provide individual and family therapy to comply with the demand of services contribute to limited access to specialized ED care [7].

In recent years, virtual interventions have become increasingly common, albeit mostly in a research context. The types of virtual treatment in place for EDs are varied and include the delivery of therapy remotely in real-time (e.g., videoconferencing), the provision of support between therapy sessions (e.g., email, texting, instant messaging), and self-help and self-monitoring interventions (e.g., internet self-help, internet-based CBT (iCBT), smartphone applications, etc.), each of which can be used alone or in combination [8, 9].

Several reviews have summarized and evaluated the evidence for psychological interventions delivered virtually for the treatment of EDs. A review on Technology-Based Interventions (TBIs) for EDs from 2013 [10] concluded that internet-based treatments are superior to waitlists for reducing ED symptoms. However, these results cannot be generalized to youth and adolescents as only one included study (of 21 studies in total) involved females in this age category. Additionally, no studies on therapy delivered via videoconferencing could be included. Given the increasing ubiquity of this modality, a new review may be warranted. A 2015 review [11] had similar conclusions but similarly included only a limited number of studies carried out exclusively in youth meeting threshold for ED diagnoses.

An updated review published in 2016 [12] suggested that iCBT and guided self-help reduced ED psychopathology, that virtual interventions may help to reach underserved populations and improve access to care, and that smartphone apps are increasingly popular but still have unknown clinical effectiveness. These results may also be somewhat difficult to generalize to youth in particular, as the review does not systematically include and focus on a specific participant age range. Though another informative and more recent paper was published in 2020 [13], studies published during the COVID-19 pandemic have not been reviewed. Furthermore, the 2020 review was not youth-specific and focused solely on RCTs, which are important for understanding effectiveness but may provide limited information about patient experience with TBIs. Additionally, reviews to date do not clearly address the extent to which interventions are delivered in a hybrid or fully online format.

More recently, the COVID-19 pandemic has brought new challenges for individuals with EDs and their care, including difficulties in accessing timely medical and psychological care due to limited services and increased demand [14]. The new constraints have led to a rapid shift to virtual care for many ED treatment programs [15, 16] and forced clinicians to rapidly adapt their methods to maintain services. The first few studies describing online adaptations made to ED care in the context of the COVID pandemic indicated general acceptability by individuals with EDs and their families as well as the overall usefulness of telemedicine [15–18]. This transition has shed new light on the use of TBIs and their potential benefits for ED treatment [15, 18], including increasing access to treatment, which will remain pertinent even after the COVID-19 pandemic.

Given the rapid changes and improvements made to virtual care modalities for EDs in the past decade, there is a pressing need for current and comprehensive studies to guide and inform clinicians in the adoption of TBIs for youth with EDs. The aim of the present study was to update existing reviews [10–13] by presenting a scoping review of research on TBIs for youth with EDs. Specifically, the objectives of this scoping review were to describe the extent to which different types of technologies have been implemented in the treatment of individuals meeting clinical diagnostic criteria for EDs, including hybrid and fully online treatments and to review the effectiveness of such interventions.

## Methods

### Search strategy

A protocol was developed but was not registered. A literature search was conducted between June 18, 2021 and February 9, 2022 using the following databases: Medline, PubMed, PsychINFO, and Google Scholar. The reference lists of relevant articles were also examined to extract additional articles. We designed and conducted the searches on the above-mentioned databases for the last 10 years (2011–2021, used as a filter). This specific time frame was chosen with the rationale that technology used before 2011 might not be representative of what is available and commonly used in more recent years and other studies [10, 11] have already reviewed the literature published before this date. Searches consisted of a combination of keywords grouped into four categories which were combined with the “AND” function: (1) telemedicine OR telehealth OR telecare OR ehealth OR e-therapy OR internet-based OR online OR web-based OR smartphone OR text messaging OR mobile OR videoconferencing; (2) eating disorder OR anorexia OR bulimia; (3) adolescen* OR child* OR young adult* and; (4) intervention OR program OR trial OR therapy OR treatment OR psychotherapy OR self-help. Note that the search engines expanded the term “eating disorder” to all related feeding and eating disorders subtopics. All fields (e.g., titles, keywords, abstracts) were searched.

### Selection process

We followed the Preferred Reporting Items for Systematic Reviews and Meta-analyses (PRISMA-ScR) guidelines for scoping reviews [19].

#### Screening 1: titles

**Fig. 1:**
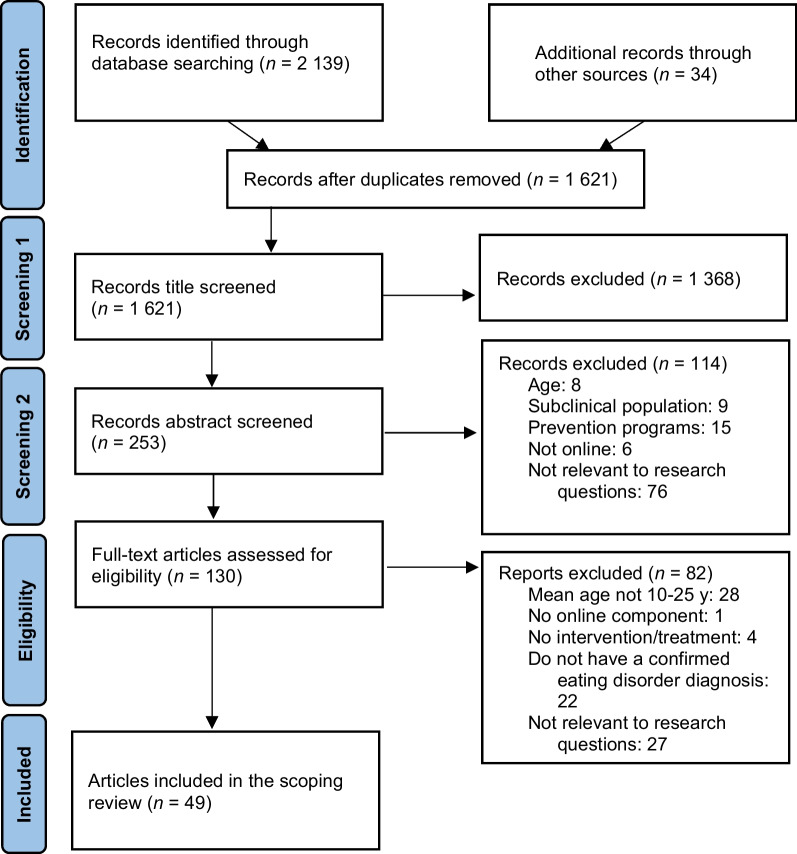
Search flow diagram

Once the records were generated from each database search, they were combined to remove duplicates. Two reviewers (RD and KN) then each screened half of the records using the titles only to determine if they were relevant or not. Titles that hinted at online interventions for EDs were retained and, to be conservative, titles that were vague were automatically moved to the next round of screening (abstract screening) (Fig. 1).

#### Screening 2: abstracts

To allow for further narrowing of the generated search records, a second screening based on abstracts was done using articles retained from screening 1. Two reviewers (RD and KN) then each screened half of the records based on their abstract. Abstracts that mentioned relevant elements of the eligibility criteria were retained for full-text review (Fig. 1).

#### Eligibility

The full texts of the remaining articles were extracted. To assess eligibility, the full text articles were independently read and rated on each of the criteria by the joint first authors of the paper (RD and KN). Discrepancies were discussed between the two reviewers. If consensus could not be reached, the two senior authors (NC and LB) made a final decision. This happened on five instances (e.g., study describing a new technology that is very different from other studies found).

Eligibility criteria were the following: (1) quantitative, qualitative, or descriptive studies published in English, French, Spanish, German, or Dutch; (2) including participants with a mean age between 10 and 25 years; (3) meeting DSM-IV or DSM-5 (or equivalent) criteria for any eating disorder, including diagnoses made using patient interviews or self-report questionnaires; (4) primarily examining TBIs (corresponding to online therapy, online support, or online self-help), and; (5) relevant to the research questions. The age cut-off of 25 years-old was based on clinical recommendations and treatment guidelines showing that the 18- to 25-year-old age group is distinct developmentally from older adults and present health disparities due to the transition from pediatric to adult health services that is often experienced during this period [20, 21]. Additionally, there is considerably less research focusing on the 10–18 years old group (*n* = 18 of 49 studies). Articles were excluded if: (1) they were published before 2011; (2) they constituted commentaries, editorials, or reviews and book chapters of studies already included, or: (3) they examined interventions addressing obesity or preventive interventions (e.g., in youth without a formal ED diagnosis). The decision to exclude studies evaluating early interventions in youth with pre-clinical/pre-diagnostic symptoms was made to facilitate the generalizability of our findings to clinical ED programs providing care to youth with a confirmed ED diagnosis. For studies using the same study sample, all relevant papers that met all criteria were retained in the full article count and Table 1, with only one being included in the quality assessment results. This was done to ensure full representation of the work done in this research area. Additionally, we made the decision a priori to include research protocols to provide the fullest understanding of the types of technology-based interventions used in the treatment of EDs in youth. This allowed us to potentially review and discuss new modalities for which no evidence yet exists but may be the object of future research.

**Table 1:** Characteristics of studies included in the review

References	Study design	Study population	Race, ethnicity, and SES	Description of intervention	Mode of delivery	Level of support	Outcome measures	Results (effect size if available)
*Empirical studies testing virtual care interventions in youth with eating disorders*								
Aardoom et al. [12, 22]	RCT	*N* = 273 Mean age = 24.4 (range not reported, over 16) Sex = 99% females Diagnosis = approximated DSM-5 diagnosis using EDE-Q self-report; 97.6% above clinical significance cut-off Recruited = via Featback website of Dutch recovery e-community **The Netherlands**	NR	Featback, a fully automated Internet-based monitoring and feedback intervention was offered alone and with additional high- and low-intensity online therapist support in comparison to a waiting list control	Online: depending on condition, all online including for therapist support	2 of the 4 conditions include email, chat, and Skype support from therapists	SEED	Featback was superior to waitlist control for bulimic psychopathology (*d* = 0.16) as well as for ED-related quality of life at 3 month follow up (*d* = 0.22) No effect for anorexia nervosa psychopathology Additional support significantly enhanced patient experience of treatment but did not impact effectiveness
Aardoom et al. [23]	RCT	*N* = 273 Mean age = 24.4 (range not reported, over 16) Sex = 99% females Diagnosis = approximated DSM-5 diagnosis using EDE-Q self-report; 97.6% above clinical significance cut-off Recruited = via Featback website of Dutch recovery e-community **The Netherlands**	NR	Featback, a fully automated Internet-based monitoring and feedback intervention was offered alone and with additional high- and low-intensity online therapist support in comparison to a waiting list control	Online: depending on condition, all online including for therapist support	2 of the 4 conditions include email, chat, and Skype support from therapists	SEED	Featback was superior to usual care for mild/moderate bulimic but not for mild/moderate symptoms of anorexia nervosa Fully automated monitoring and feedback improved mild to moderate bulimic symptoms. Additional therapist support did not increase effectiveness for these patients
Anastasiadou et al. [24]	Multicentre RCT	*N* = 106 Mean age = 18.06 (*SD* 6.04) Diagnosis = any ED, according to DSM-5 criteria Recruited from public/private mental health services **Spain**	NR	Standard CBT + TCApp, a mobile health intervention including monitoring and chat with therapists (12 weeks) compared to standard CBT alone	Hybrid: included both in-person CBT and the online mobile app in between appointments	Online chat support by therapist (therapist responded minimally once per week)	EDE-Q and SEED	There were significant but negligible reductions in EDE-Q (*d* = 0.11) and SEED scores (AN severity index: *d* = -0.09; BN severity index *d* = 0.09) for participants overall No significant difference between the 2 groups on longitudinal EDE-Q total and subscales and in severity of SEED
Anderson et al. [25]	Case series	*N* = 10 Age 13–18 years (mean = 16.08, *SD* 1.99) Sex = 8/10 were females Diagnosis = AN or atypical AN, according to DSM-5 criteria Recruited from medical ads **USA**	Ethnicity: all Caucasian SES, race: NR	Videoconference delivered Family-Based Treatment consisting of weekly family meetings with therapist (20 sessions over 6 months)	Online: intervention delivered completely via telehealth platform	No additional support outside of primary treatment	%mBMI, EDE	Weight increased from baseline to end of treatment (*d* = 0.53) and to 6 months follow-up Changes in global EDE and subscales were significant at end of treatment (*d* = 1.06) and follow-up
Carrard et al. [26]	Uncontrolled trial	*N* = 127 Mean age = 24.7 (18–43 range, *SD* = 5.1) Sex = female Diagnosis = BN purging type or EDNOS bulimic type, according to DSM-IV criteria Recruited from multiple European treatment centers and ads **Switzerland, Spain, Sweden, and Germany**	NR	Internet self-help program based on classical CBT principles	Online: all modules and contact with coach via internet. 1 evaluation session done face to face	Weekly email contact with coach	EDI-2, SCL-90R	Severity of eating disorder symptoms and general psychopathology improved significantly. 45% of participants were considered clinically improved and 23% had no symptoms at end of self-treatment program
Fichter et al. [27]	RCT	*N* = 258 Mean age 23.8 (> 16 years old, *SD*: 6.5) Sex = female Diagnosis = AN or subthreshold AN without the requirement of amenorrhea according to DSM-IV criteria Recruited from 1 of 8 hospitals in Germany specialized for ED treatment **Germany**	Ethnicity: NR SES: middle/upper class	Internet-based relapse prevention for AN after inpatient treatment using CBT strategies (9 months) compared to no intervention	Online: all components were online (chapters, electronic messaging, online chat room, automatic electronic messages)	Electronic message board for peer support + monthly 1 h chat session with therapist + therapist available via email at any time within 24 h	BMI	Patients in relapse prevention gained significantly more weight (0.62 BMI points) than patients in TAU condition (0.03 BMI points) (small to medium effect sizes for BMI change in each group from T1 to T2) EDI-2 total score showed greater decrease in TAU group over time from T1 to T2
Fichter et al. [28]	RCT	*N* = 210 Mean age 23.95, > 16 years old Sex = female Diagnosis = AN or subthreshold AN without the requirement of amenorrhea according to DSM-IV criteria **Germany**	Ethnicity: NR SES: middle/upper class	Internet-based relapse prevention for AN after inpatient treatment using CBT strategies (9 months) compared to no intervention	Online: all components were online (chapters, electronic messaging, online chat room, automatic electronic messages)	Electronic message board for peer support + monthly 1 h chat session with therapist + therapist available via email at any time within 24 h	BMI	There was an overall increase in BMI after the intervention, with a greater increase in the “full completers” group compared to the “partial completers” and the control group. There was also a significant interaction of group by time
Giel et al. [29]	Uncontrolled trial	*N* = 16 Mean age 21.7 (*SD* 4.3) No details on gender Diagnosis = full or atypical AN according to ICD-10 Recruited from clinic after inpatient care when transitioning to outpatient care **Germany**	NR	Relapse prevention program based on Maudsley Model of Anorexia Nervosa Treatment delivered by videoconference (10 sessions over 4 months)	Hybrid: 2 sessions face to face, 8 other sessions via videoconferencing	No additional support outside of primary treatment	Feasibility/acceptability; BMI, EDE-Q	Large effect size for BMI increase after treatment (*r* = 0.50), small to medium effect sizes for reduction of ED symptoms and scores (*r* range = 0.06 to 0.57)
Kim et al. [30]	Uncontrolled trial	*N* = 22 Sex = not specified Mean age = 25.27 (*SD* 8.53) Diagnosis = AN confirmed using SCID-5 according to DSM-5 criteria Recruited = from outpatient clinic of Seoul Paik Hospital **South Korea**	NR	TAU in-person (either MANTRA, specialist supportive clinical management, or FBT) augmented by videos about ED recovery tips + daily text messages + 1 weekly face-to-face guidance meeting	Hybrid: vodcast + text-message support + face-to-face therapy	Daily text message from researcher + weekly face-to-face guidance with researcher	Feasibility/acceptability; EDE-Q	Significant reduction in global EDE-Q score (*Δη*2 = 0.59) and all subscales. No significant change in BMI
Lock et al. [31]	Multicenter RCT	*N* = 40 Sex = 34 female (85%) Mean age = 14.88 (range 12–18 years, *SD* 1.81) Diagnosis = DSM-5 criteria for AN Recruited = through clinics, hospitals, and online advertisements **Canada, USA**	Ethnicity:85% white, 5% Asian, 20% other SES: majority high income	Comparison of online guided self-help family-based treatment for parents of children with AN and family-based treatment delivered via videoconferencing	Online: self-help modules were completely internet-based. Family-based treatment was delivered fully via videoconferencing	Manualized guidance provided by a “coach” therapist (avg 3.90 h over 4–6 months). For videoconferencing group: no additional support outside of online therapy sessions	%EBW, EDE	In the videoconference arm, participants gained an average of 8.63 percentage points of EBW (*Cohen's d* = 1.46) and in the guided self-help arm, participants gained an average of 9.97 percentage points (*Cohen's d* = 1.51) The EDE Global score decreased on average from 2.94 to 1.55 (*Cohen's d* = 1.04), with roughly equivalent reductions noted across both treatment arms (videoconference: 2.89 to 1.56; *Cohen's d* = 1.03; guided self-help: 2.99 to 1.54; *Cohen's d* = 1.01)
Marco et al. [32]	RCT	*N* = 34 Mean age = 21.82 (range of 15–40, *SD* 5.75) Sex = females Diagnosis = BN, EDNOS, or AN, according to DSM-IV-TR Recruited from outpatient program for EDs **Spain**	NR	CBT for EDs (15 sessions) augmented by CBT for body image in EDs using virtual reality (8 psychotherapy sessions with VR techniques)	Hybrid: all components were technically in person, although the VR is a technology that could be done online now	No additional support outside of primary treatment	Body image (BAT, BIATQ, BASS, SIBID, BITE, EAT)	All participants significantly improved body image (*η*^2^ = 0.35, corresponding to a large effect size). Effect maintained during 1-year follow-up Those with VR showed more improvement than those without VR
Neumayr et al. [33]	Pilot RCT	*N* = 40 Sex = female Mean intervention group = 20.75 (range 15–36 years, *SD* 6.4), mean control group = 18.00 (range 15–30 years, *SD* 3.73) Diagnosis = primary AN diagnosis, according to ICD-10 criteria Recruited from inpatient treatment centers for EDs **Germany**	NR	Therapist-guided smartphone-based aftercare (Recovery Record) + TAU vs. TAU alone	Hybrid: therapist-guided use of smartphone app w/in-app therapist feedback + face-to-face therapy (TAU)	Once or twice weekly in-app feedback from therapist	EDE-Q	No significant difference in BMI or EDE-Q (intervention vs. control group) from baseline to postintervention and from baseline to follow-up From baseline to postintervention: nonsignificant between-group differences of moderate effect size on EDE-Q global score (mean difference, intervention group = -0.06; control group = 0.41, *d* = 0.56)
Sanchez-Ortiz et al. [34]	RCT	*N* = 76 Sex = 75 females, 1 male Mean age = 23.9 (*SD* 5.9) Diagnosis = DSM-IV criteria for BN or EDNOS Recruited = from higher education institutions through university email addresses, posters, and pamphlets **United Kingdom**	Ethnicity: 60% British, 40% other SES: NR	Internet-based CBT with email support vs. waiting-list control	Online: internet CBT + email support	Email support from therapists ever 1–2 weeks	EDE	Differences between the internet CBT and the waitlist/delayed treatment groups increased significantly from baseline to 3 months on two of the three primary outcome variables (i.e., the EDE Global score and binge eating) EDE Global score improved in internet CBT group (*d* = 1.28) from baseline to end of treatment and to a lesser extent in the waitlist/delayed treatment group (*d* = 0.52)
Shingleton et al. [35]	Replicated single-case alternating treatment design	*N* = 12 Sex 10 females (83%) Mean age = 21.5 (*SD* 2.35) Diagnosis = DSM-5 diagnostic criteria for AN, subclinical AN, or BN with high dietary restraint/restriction Recruited via community and clinician referrals **USA**	NR	Face-to-face therapy (motivational interview + CBT) augmented by motivational text-messages + smartphone self-monitoring. Each participant underwent a semi-randomized sequence of text message and no text message phases. The phases were 1 week in duration and summed to 4 weeks of receiving text messages and 4 weeks of not receiving text messages	Hybrid: in-person therapy + text messages + smartphone app	Participants received individualized motivational text message at each mealtime	RMQ; EDE-Q	No significant main effects of the text messages on self-reported kilocalorie intake or dietary restraint (operationalized as percentage of EDE-Q questions answered “yes”) when covarying for baseline BMI Significant main effect of the text messages to increase precontemplation scores (*Wald chi-square* = 17.64) and to increase dietary restraint action scores (*Wald chi-square* = 14.85)
Wagner et al. [36]	RCT	*N* = 126 Sex = female Mean age for adolescents = 19.31 (range 16–21, SD 1.77) and for adults mean = 26 (range 22–34, SD 3.34) Diagnosis = DSM-IV criteria for BN purging type, EDNOS with binge-eating or purging behavior Recruited via ads **Austria**	NR	Internet-based guided self-help program based on CBT strategies (4–7 months) compared to bibliotherapy	Online: programme via internet platform + weekly email support	Weekly emails from therapists guiding program use	EDI-2	No difference in outcome between conditions. Both groups had significant improvement over time with intervention for monthly binge eating, vomiting, and fasting in both groups
Wagner et al. [36]	RCT	*N* = 126 Sex = females Mean age = 24.55 Diagnosis = DSM-IV criteria for BN purging type, EDNOS with binge-eating or purging behavior Recruited via ads **Austria**	NR	Internet-based (email) guided self-help vs. guided bibliotherapy	Online: internet-based self-help + weekly email support	Weekly emails from therapist guiding program use	EDI-2	No differences in abstinence and remission rates between the two groups For all EDI-2 scale scores, the scores decreased over time but there were no differences between the two interventions and no interaction
Wagner et al. [37]	RCT	*N* = 126 Sex = females Mean age = 24.55 Diagnosis = DSM-IV criteria for BN purging type, EDNOS with binge-eating or purging behavior Recruited = via ads **Austria**	NR	Internet-based (email) guided self-help vs. guided bibliotherapy	Online: internet-based self-help + weekly email support	Weekly emails from therapist guiding program use	BDI; GenPsy SIAB-EX; TCI-R; EDI-2; BNSOCQ	At the end of treatment, higher motivation to change, higher harm-avoidance, lower baseline frequency of binge eating and lower body dissatisfaction EDI-2 scores predicted good outcomes At follow-up, less baseline frequency of binge eating and higher EDI-drive-for-thinness scores were associated with good outcome at follow-up Higher BDI total scores and lower self-directedness scores at baseline predicted drop-out from therapy
Wilksch et al. [38]	Pragmatic RCT	*N* = 316 (220 met criteria for ED at baseline); Sex = female Mean age = 20.80 (range = 18–25 years, *SD* 2.26) Diagnosis = AN, BN, BED, or OSFED assessed using EDE-Q Recruited via self-referral by advertisements **Australia and New Zealand**	NR	Media Smart-Targeted (MS-T), an internet self-help program vs. receiving tips for positive body image	Online: internet-based self-help program without support	No support provided via app	EDE-Q	MS-T participants were 75% less likely than controls to meet ED criteria at 12-month follow-up This was statistically significant amongst both nontreatment seekers and treatment seekers who were 71% and 86% less likely than controls to meet diagnosis at 12-month follow-up, respectively
*Observational and mixed-methods studies*								
Barney et al. [18]	Observational	Set at Adolescent and Young Adult Medicine clinic Age range = 12–26 years Sex = mostly females 1715 unique patients during this time Diagnosis = any adolescent/young adult health concern, including all EDs Info also included providers, clinical support staff, clerical support staff and other health professionals **USA**	NR	Within 1 week of pandemic, all providers used Zoom for telemedicine. By week 2, daily telemedicine sessions began for urgent care, mental health, ED, and addiction treatments	Online: telemedicine clinics, daily 2 h in-person sessions for special cases only	All providers (e.g., nurses, fellows, physicians) continued their assessments and sessions online	Measured healthcare service usage and qualitative user/provider experience throughout pandemic	Telemedicine visits increased from 0 to 97% in one month, number of visits comparable to year prior. Providers identified several barriers and solutions to providing different types of care: using earphones for privacy, full training on Zoom, patients submitting results to their patient portal, training families to collect weight at home, having weight taken by nurses, etc. These were identified to help with barriers such as patients not having appropriate devices, technology literacy gap with families, lack of connection etc
Carretier et al. [39]	Case study and description of implementation of telemedicine during COVID-19 pandemic	*N* = 1 (case study) 16-year-old female teenager with restrictive AN Recruited from the Maison des Adolescents where being treated pre-COVID-19 **France**	NR	Virtual day hospital care, including individual group, and family therapy was provided by videoconference by a multidisciplinary adolescent medicine clinic	Online: all sessions moved online. for critical cases, some in-person appts possible	No additional support outside of multidisciplinary videoconference therapy	Qualitative case description and discussion	The center was able to move online quickly and efficiently. Benefits of telemedicine are discussed: teleconsultations allow to identify potential risk factors (ex. see how Anna deteriorated during the confinement), telemedicine reduces isolation of patients and allows for clinical support at home, the technology involved is mastered by adolescents and they feel comfortable using it, telemedicine can facilitate the discussion of difficult questions and help work on relationships (ex. helped Anna's family create a dialogue on ED)
Criquillion et al. [40]	Proposal for a non-randomized clinical trial	Goal is *N* = 50 patients Sex not specified Age range = between 12 and 35 years old Recruited from a clinic for EDs, patients (42 patients currently involved in the testing of the app) **France**	n/a	A psychoeducational smartphone application including real-time follow-up for use in clinical settings is proposed	Hybrid: partially virtual; followed at the medical clinic regularly, the app on the side for continuity	No support via app, although the patients are followed for the rest of their treatment by doctors at the clinic	Evolution of baseline characteristics	n/a
Datta et al. [15]	Observational	Description of patients seen at the clinic: *N* = 1582 82% female, 17% male, 1% non-binary Mean age = 16.5 (age range up to 25 years, *SD* 1.72) Diagnosis = mostly AN but included all EDs, as diagnosed by physicians in the specialized ED clinic Recruited from a comprehensive care unit for EDs **USA**	NR	Several parts of treatment were delivered via videoconference: 1) psychotherapy and medication management 2) therapeutic groups based on CBT, cognitive remediation therapy (CRT), process group (PG) and parent/patient tele-groups 3) new admission evaluations	Online: mostly videoconferencing for therapy session and group work. When necessary, some in-person	All online aspects of treatments were provided by clinicians: psychologists, psychiatrist, and dietitians. No support outside of regular treatment	Qualitative descriptions of the changes made at the treatment center	All aspects of new admissions, psychotherapy, medication management, and therapeutic groups were moved online where they used videoconferencing. benefits/takeaways are these: 1) useful for parents to take part in initial evaluations remotely 2) remote work generates exposures for social anxiety triggers and complaints 3) videoconferencing useful for bed restriction patients 4) setting group rules is possible and helps when conducting remote group sessions
Davis et al. [16]	Observational	No sample population Description of Singapore hospital multidisciplinary ED service for patients 9 to 16 (mean 13.9, *SD* 1.5) **Singapore**	NR	For inpatient: individual meal supervision by nurses. For outpatient: telehealth instituted for physicians and psychologists to manage selected cases and for ongoing engagement with families by psychologists	Hybrid: mix of in person and telehealth	No additional support described outside of videoconference therapy	n/a	The paper describes several considerations for use of telemedicine: for patient/family: willing to use telemedicine, weight stable or good consistent progress, parent willing to weigh patient, no safety concerns, no concerns of medical stability. for health care provider: develop patient eligibility criteria for telemedicine, undergo training in telemedicine
Kasson et al. [41]	Mixed method	(a) *N* = 14 Sex = 100% female Age range = 14- 17 years Diagnosis = clinical or subclinical ED according to DSM-5 criteria, based on self-report questionnaire (excluding clinical AN) Recruited via Instagram & Facebook (b) *N* = 30 Sex = 13% gender minority, 74% female, 13% male Age range = 14 – 17 years Diagnosis = same as above Recruited via Instagram, Facebook, Snapchat, YouTube, & TikTok **USA**	(a) 92% White; 8% Other Hispanic 14%; Non-Hispanic 86% 71% sexual minority (b) 74% White; 26% Other 13% Hispanic; 87% Non-Hispanic 57% sexual minority; 63% LGBTQ + group	Mobile phone and desktop application, *Space from Body and Eating Concerns Program,* based on CBT for EDs	n/a	n/a	Stanford-Washington Eating Disorder Screen	Using social media for recruitment of teens with EDs is feasible and may help in capturing more diverse samples The youth who participated in the survey were highly interested in a mobile app to help with ED recovery
Rajankar et al. [42]	Intervention description	**India**	n/a	Proposal for a new smartphone application combining awareness of etiology/informational section, health tracker, calorie tracker, virtual intervention (CBT, DBT, Self-help, support groups), and alert function notifying nearby NGOs/helplines	Smartphone app	n/a	n/a	n/a
Shaw [43]	Observational	*N* = 3 Sex = female Age range = 12–17 years old Diagnosis = AN, as diagnosed by treating physicians in a specialized ED clinic Recruited from an eating disorder treatment service **United Kingdom**	NR	7-week video-conference art therapy sessions held on Microsoft Teams platform	Online: videoconference	No additional support outside of videoconference therapy	Qualitative description of experience	Challenges when working with this client group online include increased body image concerns changed experience of gaze
Stewart et al. [44]	Mixed methods	*N* = 53 Age range = < 18 years old Diagnosis = AN, AAN, BN, ARFID, or other specified feeding and eating disorder, as diagnosed by treating physicians in the specialized ED clinic Recruitment = all clinicians, young people, and parents working or receiving treatment at an outpatient community ED program for youth during data collection were eligible **United Kingdom**	NR	Experience in response to COVID-19 pandemic changes: all but essential face-to-face contact was ceased, all outpatient treatment was delivered by video/phone calls	Online only (besides essential services)	No additional support outside primary treatment from family therapists, psychiatrists, nurse therapists, clinical psychologists	Questions about clinician, young people, and parent experience of providing/receiving online treatment	Young people (YP) and parents had overall positive experience with online therapy, low level of impact of technology on treatment experience; Qualitative Themes: Gratitude/something lost/something gained; home as a therapeutic space; changes in therapeutic relationship; implications for the future; trade-off between increasing and limiting access by moving online
Raykos et al. [45]	Uncontrolled trial	*N* = 25 Sex = 93% female Mean age = 24.4 (*SD* 7.6) Diagnosis = any ED, according to DSM-5 criteria, using EDE & SCID-5 Recruited from patients being followed in an outpatient ED clinic **Australia**	70% Anglo/European-Australian	Rapid transition from face to face to telehealth care in an outpatient clinic for EDs, in the context of COVID-19. FBT or CBT was delivered via telehealth, based on age and diagnosis	Online: telehealth	No additional support provided outside regular treatment	EDE-Q, ED-15, CIA, BMI,	Throughout the study period, patients experienced a large decrease in ED symptoms, comparable to historic benchmarks at the clinic (from pre- to during COVID-19, EDE-Q *d* = 1.62) Patients rated the quality of treatment and therapeutic alliance highly
Tregarthen et al. [46]	Observational	*N* = 108,996 Sex = 57,940 provided gender (87.2% female) Mean age = 22 (reported by 48,830, range = 13–77 years) Diagnosis = symptom severity assessed by EDE-Q, 87.2% were ≥ 1 *SD*s above community norms Recruitment = app was made available for download in app stores	NR	CBT-based self-monitoring smartphone application	Online: smartphone app	No support provided via app	Acceptability; EDE-Q, BMI at baseline	Acceptability: 2,503 anonymous ratings: 84% were 5/5, 13.3% were 4/5, 1.7% were 3/5, and 1% were 2/5 BMI (calculated for 13,784 users): 10.6% met the BMI suggested weight criterion (< 17.5) for a diagnosis of anorexia nervosa Any occurrence of objective binge episodes 78.8%, subjective binge episodes 76.79%. self-induced vomiting 41.9%, fasting 63.3%, excessive exercise 59.7%, laxative misuse 20.6%
Yaffa et al. [47]	Case series	*N* = 4 Sex = female Age range = 13–17 years Diagnosis = any ED Recruitment = patients were receiving services at a specialized pediatric ED treatment center in a children’s hospital **Israel**	NR	Management of pts in the context of COVID-19 pandemic and lockdown	Online: treatment via videoconferencing; in-person visits occurred only in specific conditions	No additional support outside videoconference treatment provided by psychiatrist, clinical nutritionists, psychotherapists	-	The condition of the four adolescents with AN was compromised at the start of the COVID-19 quarantine. The use of multi-disciplinary long- distance telemedicine treatment resulted in an improvement in the condition in three of the four adolescents, living in well-organized families, with the motivation and ability to adjust to the new conditions, but not in one adolescent whose family experienced more problems
*Qualitative studies*								
Anastasiadou et al. [48]	Qualitative study	1) *N* = 8, health care providers and mobile health experts, mean age 34 (*SD* 7.21), psychiatry, psychology and nursing, males and females 2) *N* = 9 participants of RCT Mean age = 15 (*SD* 0.50) Sex = all females Diagnosis = any ED, according to DSM-5 criteria Recruited from RCT participants [24] **Spain**	NR	Standard CBT + TCApp, a mobile health intervention including monitoring and chat with therapists (12 weeks)	Hybrid: included both in-person CBT and the online mobile app in between appointments	Online chat support by therapist	Questionnaire assessing services based on effectiveness, user experiences, economic aspects, organizational aspects, sociocultural aspects	Key themes identified by specialists: lack of time, lack of strategic plan, lack of budget, insufficient training, lack of tech support, lack of familiarity and mobile health skills, lack of agreement, insufficient interaction, security/privacy issues. Perceived advantages by patients: ease of use, useful, liked the app design, content appropriate. Perceived disadvantages: problems with design, lack of satisfaction with personalization, some say limited interaction
Brothwood et al. [49]	Qualitative study	14 patients and 19 parents Age range = 12 -18 years Mostly females, one male Diagnosis = AN-R, according to DSM-5 criteria Recruited via day program at Maudsley center for child and adolescent ED in London **United Kingdom**	NR	During the COVID-19 pandemic, day treatment program based on principles of family therapy was delivered online. Online activities included therapy, groups, meal support, and education support. All kinds of therapy, exercises, worksheets, and coaching for meal support, were provided online	Online: treatment online. Urgent appts offered in person if increased physical or psychiatric risks	Mix of clinician support for groups, therapy, and meals. Some exercises were self-help online	User experience questionnaires	Overall experience was positive (slightly more so for parents), quality partly impacted, parents felt more comfortable, young people reported issues with technology. But majority thought could not have done anything differently. Each component rated by majority as somewhat or very helpful. Compared to face to face, varied response but found less helpful (specifically meal support, family sessions and individual sessions). Young people rated that they want those to be in-person in future but would keep medication reviews or dietetic appts virtually. Qualitative data themes: 1) new discoveries 2) lost in translation 3) best of bad situation
Lindgreen et al. [50]	Qualitative study	*N* = 41 Mean age = 24.0 (age range = 15–41 years, *SD* 5.9) Sex: 3 male, 38 female Diagnoses = anorexia nervosa and bulimia, as diagnosed by clinicians at the specialized ED treatment facility Recruited = from a specialized ED treatment facility **Denmark**	NR	Treatment as usual (for AN: family-based treatment or weekly group/individual sessions; for BN: 10 weekly manualized group sessions followed by an additional group or individual treatment) augmented using Recovery Record (RR) self-monitoring app for eating disorders	Hybrid: in-person sessions augmented by guided self-monitoring smartphone app use	Therapists provided guidance on app usage during in-person therapy sessions	User experience with Recovery Record app	Patient experience with the app depended on its app features, the impact of these features on patients, and their specific app usage. This patient-app interaction affected and was affected by changeable contexts making patients' experiences dynamic
Naccache et al. [51]	Qualitative study	*N* = 8 Mean age = 15.5 (age range = 12–18, *SD* 1.07), Sex = all females Diagnosis = AN, according to DSM-5 criteria All enrolled in treatment at Toulouse Teaching Hospital **France**	NR	Unguided self-help program app: based on psychoeducation, CBT, and motivational interview strategies. Additional emotional management feature and gamification elements	Online: testing only user experience of the smartphone app	No additional support in relation to the app	UEQ and semi-structured interviews	Patient experience of the app was demonstrated with key themes, including finding coping strategies, information, motivational content helpful. Overall, the app was rated positively, although opinions were mixed regarding novelty, dependability and efficiency. Qualitative data showed that patients and clinicians think the psychoeducation portion is important
Sanchez-Ortiz et al. [52]	Qualitative study (recruited as part of an RCT)	Interview: *N* = 9 Sex = female Mean age = 23.2 (*SD* 3.5) Diagnoses of BN or EDNOS-BN Questionnaire: *N* = 31 All participants were drawn from those participating in RCT on internet CBT and were students recruited from university networks [34] **United Kingdom**	NR	Internet-based CBT program called "Overcoming bulimia online" comprised of immediate iCBT with email support from therapist (4 to 8 sessions)	Online: intervention provided completely online via platform + email	Therapist support via weekly emails	Semi-structured and questionnaires on iCBT treatment experience	Experiences of treatment were summarised in five categories: confidentiality/privacy, flexibility, ease of use, feeling supported, and content of programme. As for impact of treatment, overall positive impression, categorized into: (1) expectations about outcome, (2) effectiveness – reported change in ED symptoms, (3) effectiveness—other changes, and (4) tools for coping in future. As for all RCT participants impressions: struggled to find motivation to continue treatment, knowledge and confidence grew, most useful aspects = weekly emails, problems with access to treatment/technical difficulties, most common suggestion = more support and some face to face*
Shaw et al. [53]	Qualitative	*N* = 43 participants; 19 parents/carers, 12 members of staff, 12 patients, all < 18 years of age Diagnosis = typical or non-typical AN, BN, or BED recruited from a hospital ED service **United Kingdom**	NR	Describes experience of adaptations made in response to COVID-19 pandemic	Hybrid: in-person physical examinations with all other appointments online	No additional support outside of primary treatment	Healthcare service usage (hospitalizations, referrals, admissions); user satisfaction, narrative analysis	More referrals accepted in 2020 than 2019, higher % increase for urgent vs. routine consultations (150% vs 129%, respectively); Qualitative results: Using virtual platforms improved ease of access but altered relational experiences
*Case studies*								
Hellner et al. [54]	Case series	*N* = 2 Female Age = 20 and 15, respectively Diagnosis = AN or atypical AN Recruited by Equip providers through postings **USA**	NR	Videoconference delivered multidisciplinary treatment based on FBT, CBT, and DBT. Patient met with each provider weekly or more	Online: all done through telehealth and unlimited messages on the telehealth platform	Team support (family therapist, dietitian, peer mentor and family mentor) via chat	Weight gain, EDE-Q short form	Both patients gained at least one pound per week. Scores for EDE-Q decreased by 7–12 point
Duncan et al. [ 55]	Case study	*N* = 1 Sex = female Age = 14 years Diagnosis = EDNOS Recruited from rural tele-psychological clinic in her community **USA**	NR	Videoconference delivered therapy, based on cognitive behavioral strategies	Online: all sessions done over videoconferencing platform. 1 appointment was done in person	No additional support outside of primary treatment	n/a	At the end of the 12 sessions of therapy, Mila increased food intake, fell within normal range on growth chart, and scores on CDI no longer elevated
Study protocols*								
Anderson et al. [56]	Case series	*N* = 10 (target) Age range = 13–18 years Sex = no specification Diagnosis = DSM-5 criteria for AN or atypical AN Recruited = by referrals and through community **USA**	NR	Videoconference delivered Family-Based Treatment consisting of weekly family meetings with therapist (20 sessions over 6 months)	Online: intervention delivered completely via telehealth platform	No additional support outside of primary treatment	Feasibility/Acceptability of FBT via Telemedicine	Protocol only
Barakat et al. [57]	Multi-site three-arm RCT	*N* = 110 (target) Gender will be reported Age range ≥ 16 years Diagnosis = DSM-5 criteria for BN or OSFED with bulimic behaviors Recruited = from general population via advertisement and referrals from health professional **Australia**	Income, and cultural background/ethnicity will be reported	Online CBT-based self-help program completed independently vs. completed with therapist guidance (= weekly telemedicine sessions) vs. waitlist control	Online: either online self-help platform or secure videoconference platform	For self-help group with guidance only: weekly 30 min videoconference session with therapist	Frequency of objective binge episodes (EDE-Q)	Protocol only
Bulik et al. [58]	RCT	*N* = 180 (target) Sex: no specification Age range ≥ 18 years old Diagnosis = criteria for DSM-IV BN Recruited from within local health services and via ads **USA**	NR	Group CBT delivered via the internet versus face-to-face	Online: online modules with text-based chat group (without audio/video)	Therapist-led chat groups or in-person therapist support in groups	EDE	Protocol only
de Zwaan et al. [59]	Multicenter RCT	*N* = 178 (target) Age range ≥ 18 years old Diagnosis = DSM-IV criteria for BED or subsyndromal BED Recruited = via ads **Germany and Switzerland**	NR	Internet-based guided self-help vs. individual in-person CBT	Hybrid: 2 in-person meetings with therapist + internet-based email-guided self help	Therapist support by email	Difference in number of days with OBEs over the past 28 days (assessed by EDE)	Protocol only
Hambleton et al. [60]	Uncontrolled trial	Goal is 41 families Age range = 12–18 years Gender will be reported Diagnosis = criteria for AN according to DSM-5 Recruited from regional or rural health district via referral **Australia**	SES will be reported	Family-based treatment delivered via videoconference (18 session over 9 months) via telemedicine	Online: via videoconferencing platform (or via telephone if technical difficulties)	No additional support outside of primary treatment	Remission (increase in %mBMI to ≥ 85%)	Protocol only
Jenkins et al. [61]	RCT	*N* = 51 (target) Sex not specified Age range ≥ 17.5 years Diagnosis = regular binge eating in the context of an ED Recruited = clinical referrals **United Kingdom**	NR	Self-help delivered face-to-face versus via e-mail versus delayed treatment control condition	Hybrid: first guidance session provided in-person	Therapist support via email, up to twice weekly	Frequency of objective bulimic episodes assessed by EDE-Q	Protocol only
Kolar et al. [62]	RCT	*N* = 30 (target) Sex = female Age range = 12–19 years Diagnosis = AN **Germany**	NR	In-person consultations with smartphone app vs. in-person consultations alone	Hybrid: in-person therapy + smartphone app use	Therapist support in-person	Weight gain	Protocol only
Kolar et al. [63]	Multi-center RCT	*N* = 30 (target) Sex = female Age range = 12–19 years Diagnosis = AN or atypical AN confirmed by EDE structured interview Recruited = from waiting list from 3 child & adolescent psychiatry centers **Germany**	NR	Face-to-face supportive psychiatric follow-up augmented by therapist-guided use of a DBT-informed smartphone application, (Jourvie) vs. TAU (supportive therapy by psychiatrist)	Hybrid: face-to-face therapy + guided smartphone app use	In-person therapy sessions will include guidance on use of the smartphone app	BMI-SDS, EDI-2	Protocol only
Rohrbach et al. [64]	RCT (two-by-two factorial design with repeated measures)	*N* = 352 (target) Sex not specified Age range ≥ 16 years Diagnosis = at least mild self-reported ED symptoms on standardized questionnaire Recruitment = community Dutch e-community ED website **Netherlands**	NR	(1) Featback, a fully automated self-guided Internet-based monitoring and feedback intervention augmented by weekly chat/email support from an expert patient was compared to (2) Featback without expert patient support, (3) expert patient support only, and (4) waiting list	Online: unguided internet psychoeducation with or without chat/email support	Weekly expert patient support via chat and email (in 2/4 conditions), no additional support in 2/4 conditions	EDE-Q	Protocol only
Schlegl et al. [65]	RCT	*N* = 186 participants (target) Sex = female Age range = 12–60 years Diagnosis = diagnosis of AN at hospital admission, according to DSM-5 criteria Recruited = at an ED clinic **Germany**	NR	Therapist-guided smartphone-based aftercare (Recovery Record) + TAU vs. TAU alone	Hybrid: therapist-guided use of smartphone app w/in-app therapist feedback + face-to-face therapy (TAU)	Psychotherapists provide individual feedback via-in app messages	EDE	Protocol only
ter Huurne et al. [66]	RCT	*N* = 252 (target) Age range ≥ 18 year Sex = female Diagnosis = DSM-IV criteria for BN, BED, or EDNOS Recruited = self-selection via targeted ads **The Netherlands**	NR	CBT-based internet self-help compared to waiting list control group	Online: intervention and therapist support via online treatment platform	Therapist support by email twice weekly; Forum for peer support	EDE-Q	Protocol only

*NR* not reported, n/a not applicable, *ED* eating disorder, *AN* anorexia nervosa, *AAN* atypical anorexia nervosa, *AN-R* anorexia nervosa restricting type, *ARFID* avoidant/restrictive food intake disorder, *BN* bulimia nervosa, *BED* binge-eating disorder, *EDNOS* eating disorder not otherwise specified *OSFED* other specified feeding or eating disorders, *ICD-10* international classification of diseases 10th revision, *DSM-IV* diagnostic and statistical manual of mental disorders fourth edition, *DSM-5* diagnostic and statistical manual of mental disorders fifth edition, *SES* socioeconomic status, *CBT* cognitive behavioral therapy, *DBT* dialectical behavior therapy, *RCT* randomized controlled trial, *TAU* treatment as usual, *SCID-5*: SEED Short evaluation of eating disorders, *%mBMI* Percent median BMI, *EDE-Q* eating disorder examination-questionnaire, *EDE* eating disorder examination, *EDI-2* eating disorder inventory-2, *SCL-90R* symptom checklist-90-revised instrument, *BMI* body mass index, *%EBW* percent expected mean body weight, *BAT* body attitude test, *BIATQ* body image automatic thoughts questionnaire, *BASS* body areas satisfaction scale, *SIBID* situational inventory of body image dysphoria, *BITE* bulimic investigatory test, *EAT* eating attitudes test, *BDI* Beck depression inventory, *GenPsy of SIAB-EX* general psychopathology of the structured interview for anorexic and bulimic disorders, *TCI-R* temperament and character inventory revised, *BNSOCQ* bulimia nervosa stages of change questionnaire, *UEQ* user experience questionnaire, *BMI* body mass index, *USA* United States of America

^*^Note that we included these study protocols on the basis that they had the potential of having a sample mean age that fit our inclusion criteria considering their target population

#### Charting the data

The data charting process was carried out independently by the joint first authors of the paper (RD and KN), who each read and extracted information from half of the eligible articles. The reviewers each inputted data into a separate, dedicated spreadsheet which included the following categories: (1) sample characteristics, including: number of participants, age (mean, range, and standard deviation), race, ethnicity, socioeconomic status, and recruitment details; (2) study design; (3) a description of the intervention; (4) mode of delivery of the described intervention; (5) type of provider (e.g., psychiatrist, therapist, etc.); (6) outcome measures described in the article; (7) results, including primary and secondary outcomes in empirical studies and highlights from qualitative and descriptive studies, and; (8) key takeaways and conclusions from the study. When any of the above-mentioned information was not included in the publication, except when non-applicable due to the nature of the article/study, the corresponding author of the study was contacted by email for clarifications or to obtain the necessary results.

#### Quality assessment

A critical appraisal was carried out for quantitative and qualitative studies in order to describe the quality of the evidence included in the review. Quality ratings were not used as an eligibility criterion, as the goal of the review was to describe the widest array of studies on this topic and studies were discussed narratively. For quantitative studies, a 19-item tool adapted from Greenhalgh and Brown [67] and Higgins et al. [68] was used because it fit best with the heterogeneity of methods described in the studies. Two unblinded reviewers (RD and KN) independently assessed the articles on each item using “yes”, “no”, “not reported”, or “non-applicable”. For qualitative studies, the 10-item Critical Appraisal Skills Programme [69] checklist was used. The same unblinded reviewers independently assessed the articles on each item using “yes,” “no,” or “can’t tell.” For both the quantitative and qualitative studies, disagreements were discussed between the two reviewers until consensus was reached (see Additional file 1: Fig. S1 and Additional file 2: Fig. S2 for final ratings). Note that we did not conduct a quality assessment for observational/descriptive studies, as there was too much variability in their methodology.

### Synthesis of results

Data pertaining to articles describing intervention trials with quantitative results were compiled and placed into a table for data presentation. Those articles were analyzed based on mode of delivery of the intervention and virtual versus hybrid (in-person and virtual) delivery to answer the main research questions, using available data measures such as effect sizes. The remaining articles were categorized into qualitative/mixed-methods studies, descriptions of adaptations to the COVID-19 pandemic, and new technologies that could potentially be used in the future in the treatment of EDs.

## Results

### Identification of studies and quality assessment

The searches from the four electronic databases and reference lists led to a total of 2173 records (Medline: 491, Pubmed: 732, PsychINFO: 291, Google Scholar: 625), which was then reduced to 1621 records after removal of duplicates. A total of 130 articles were retrieved in full-text and assessed for eligibility (see Fig. 1). Finally, 49 articles met full inclusion criteria and were included in the scoping review. Of these, 29 articles used quantitative methodologies to evaluate interventions, and these were prioritized for analysis of effectiveness. The remaining 20 studies, of which 12 were observational or descriptive and eight were mixed methods or qualitative studies, were described separately (see Table 1). Overall, most qualitative and mixed methods studies (7/8) can be considered of high quality (i.e., meeting at least 8/10 criteria) according to the critical appraisal checklist (see Additional file 2: Fig. S2). The remaining study met 6/10 criteria.

Of the quantitative articles describing intervention trials (*n* = 29), 18 included results, while 11 consisted of study protocols only. Note that we included these protocols on the basis that they had the potential of having a sample age that fit our inclusion criteria considering their target population. The 18 quantitative papers covered results from 14 intervention trials, with a few papers analyzing different results from the same trial. Overall, 78% (*n* = 14) of the 18 quantitative studies were qualified as high quality/low risk of bias, meaning that more than 10 out of 19 quality assessment criteria were met (i.e., “yes” or “partially yes”), and 22% (*n* = 4) were qualified as moderate to low quality/moderate to high risk of bias, meaning that fewer than 10 out of 19 criteria were met (see Additional file 1: Fig. S1). Of those four articles, only one study met fewer than seven criteria.

Virtual modalities were generally used to deliver primary (*vs*. adjunctive) treatment. We considered interventions used both within sessions with clinicians as well as those used in between sessions by patients alone. Our review found therapy delivered via videoconference, mobile applications, and internet self-help to be the most used and each of these, in addition to other technologies, are discussed separately below. However, these modalities have also been used as adjunct interventions to increase points of contact with therapists in between appointments via email, instant-messaging, text message, telephone, or automated messages. Of the 29 intervention studies reviewed and discussed in more detail below, 20 studies (69%) provided some type of supplemental virtual support. Email contact (or asynchronous messages within applications) was most frequent (*n* = 15 studies), followed by instant-messaging support (*n* = 5 studies), automated support (*n* = 5 studies), videoconference support (*n* = 2 studies), and text message support (*n* = 1 study).

Another important component of TBIs and treatment for EDs is whether the intervention was delivered fully online or was supplemented by in-person appointments, in what could be described as hybrid interventions. Table 2 summarizes evidence for hybrid and fully online interventions by modality. Overall, there are fewer interventions that are considered hybrid. Almost all internet self-help interventions (11/12) were fully online and most mobile app interventions were hybrid (5/7). Effect sizes were largest for fully online videoconferencing interventions.

**Table 2:** Summary of effect sizes and patient experiences with hybrid and fully online interventions for the treatment of eating disorders in youth

	Total # of studies	# of quantitative studies	# of qualitative/mixed studies	Effect size	Summary of patient experiences
*Hybrid*					
Videoconferencing	3	1	2*	Large (*r* = .50)	Treatment satisfaction similar for in-person and online Some voiced concerns for therapeutic relation and had preference for in-person care
Mobile apps	5	3	2	Small to moderate (*d* = .09 to .41)	Useful, easy-to-use tool Individual experience dependent on context of use Experience could be improved by increasing flexibility of features, ability to personalize app, and by including motivational/interactive components
Internet self-help	1	1	0	Large (*Δη*2 = 0.59)	–
*Fully online*					
Videoconferencing	9	2	10* (including 2 case studies)	Medium to large (*d* = .53 to 1.51)	Can lead to improvements in eating disorder symptoms Accessibility, convenience especially appreciated by parents. More useful for motivated families Relational disconnect, technological barriers reported Concerns for confidentiality if limited private space at home In-person treatment preferred
Mobile apps	2	0	3 (including 1 case study)	–	Apps informative and acceptable for patients, clinicians Support from clinician appreciated but privacy concerns when sharing information Would appreciate more personalization, multimedia elements Patients more willing to use well-known apps, those recommended by clinicians
Internet self-help	11	10	1	Small to large (*d* = .16 to 1.28)	Rated as relevant, effective, and convenient by participants Email support essential source of motivation, though many wanted more support and other forms (in-person, phone calls) as self-motivation was difficult

*Includes studies describing the transition to online services during the COVID-19 pandemic. Effect sizes reflect range for quantitative studies

### Modes of delivery: quantitative studies

TBIs are reviewed below, based on the mode of delivery described in the study.

#### Videoconference platforms for delivery of therapy

Our search identified four articles [25, 29, 31, 56] on the use of videoconference technology for ED treatment. This included a pair of articles by Anderson et al. discussing an experimental protocol [56] and its subsequent results [25]. In all articles, the therapy provided via videoconference constituted the primary treatment, but there was variation in terms of the addition or not of in-person therapy sessions and in-person medical follow-ups.

Two of the studies [25, 31, 56] evaluated the use of videoconference for delivering FBT for individuals with anorexia nervosa (AN). One of these was a pilot, multicenter, randomized controlled trial (RCT) [31] comparing two methods for delivering FBT in a sample of 40 participants with AN (aged 12–18 years, mean 14.88 years), either via a videoconferencing platform, in which case the whole family of the adolescent participated, or via an online guided self-help program, in which case only the parents of adolescents participated. At the end of treatment, there was an overall improvement, regardless of treatment group, in ED psychopathology measured using the Eating Disorder Examination (EDE) interview Global score with a large effect size (*d* = 1.04). The change was similar in both treatment groups (videoconference: *d* = 1.03; guided self-help: *d* = 1.01). The second was an uncontrolled trial [25, 56] targeting adolescents (*n* = 10) aged 13–18 years (mean age = 16.1 years) with typical or atypical AN, consisting of 20 sessions of family-based treatment given over 6 months, during which time the therapist maintained contact with the treating pediatrician, who evaluated participants in-person. Participant weight (*d* = 0.53) and self-reported ED symptoms (*d* = 1.06) improved from baseline to end-of-treatment and moderate to large effect sizes were maintained at 6-month follow-up [25].

A third article described an uncontrolled trial by Giel et al. [29], evaluating a videoconference relapse prevention program based on the Maudsley Model of AN Treatment was evaluated. The intervention included an initial and final in-person therapy session and eight videoconference therapy sessions, given over a period of 4 months to participants (*n* = 16) with a mean age of 21.7 years (*SD* = 4.3) and a diagnosis of AN. Participants who completed the intervention (*n* = 12) showed significantly lower scores on the eating concerns subscale of the standardized eating disorder examination questionnaire (EDE-Q) post-intervention as compared to before. Though not statistically significant, effect sizes for BMI change (*r* = 0.50), body shape concerns subscale (*r* = 0.43), weight concerns subscale (*r* = 0.36), and global EDE-Q scores (*r* = 0.41) were medium to large.

#### Mobile apps

Mobile apps can be used in multiple ways for mental health interventions including as a supplement to treatment, as a self-help tool between appointments, or as a monitoring device following inpatient treatment [70]. We identified two studies which aimed to test the effectiveness of different app-based interventions.

One article described the *TCApp* mobile app. This app consists of features including self-records, food records, and thought records, monitoring, and instant messaging with the therapist. Anastasiadou et al. [24] conducted a multicentre RCT to compare the effectiveness of standard CBT augmented with *TCApp* to treatment as usual in participants with any ED diagnosis (*n* = 106, mean age = 18.06 years). The authors found no significant difference between the two groups on the EDE-Q global scale and subscales over time. Regardless of the treatment group, CBT reduced overall symptom severity with medium to large effect sizes (*r* range = 0.46–0.63) [24].

A second article described *Recovery Record*, an app which features self-monitoring, a thoughts and feelings journal, coping strategies, personalized goals, and contact with a clinician as the central elements. In a pilot RCT by Neumayr et al. [33] treatment combining the use of *Recovery Record* with treatment as usual was compared to treatment as usual alone in participants with AN (*n* = 40, age range = 15–36 years). The authors found no significant differences between baseline and post-intervention for BMI or EDE-Q, and non-significant between-group differences with moderate effect sizes for EDE-Q global scores (*d* = 0.56) and subscales (ranging from *d* = 0.33 to 0.64), and a small effect size for BMI (*d* = − 0.24), in favor of the intervention group.

#### Internet self-help

Seven manuscripts described an intervention that offered online self-help modules and these varied substantially in content and therapeutic approach.

We identified four trials testing internet-based self-help programs utilizing CBT principles such as self-monitoring, stimulus control, operant conditioning methods, exposure treatment, and cognitive restructuring [26, 27, 34, 36]. The following studies may differ in terms of the amount of internet self-help modules used and their specific content. An RCT [27, 28] focused on a 9-month web-based relapse prevention program for AN (*n* = 258, mean age = 23.8 years) following inpatient treatment with a subsequent 9-month follow-up study [28]. Participants who completed the intervention gained significantly more weight (0.62 BMI points) than those who received treatment as usual, corresponding to a small to medium effect size (mean *d *= 0.22). At 9-month follow-up from the end of treatment, weight gain remained greater in the intervention group, though the between-group difference was no longer significant [28]. Notably, while eating disorder symptoms increased in both conditions, the internet self-help group showed a lower increase in ED symptoms [27]. Another RCT by Wagner et al. [36] compared the effectiveness of CBT-based internet self-help to bibliotherapy in both adolescents and adults with binge/purge type EDs (*n* = 126, age range = 16–21 years). They found that both interventions were effective and led to significant improvement on primary outcomes, (i.e., monthly binge eating, vomiting, and fasting). Additionally, 46.5% of the participants reported being abstinent from binging for 7 months following both interventions [36]. Similarly, one RCT testing the effectiveness of iCBT-based self-help with email support in 76 participants (mean age = 23.9 years) with BN found a greater reduction in EDE global scores in the iCBT group (*d* = 1.28, corresponding to a large effect size) than in the waitlist control group (*d* = 0.52, medium effect size) [34]. Finally, an uncontrolled trial by Carrard et al. [26] examining iCBT self-help in 127 women (mean age = 24.68 years) with bulimia nervosa (BN) found that 45% of participants had clinically improved following the intervention, with significant improvements on outcomes such as binge episodes, self-induced vomiting, and over-exercising, as measured by the Eating Disorder Inventory-2.

One RCT tested the effectiveness of an internet self-help psychoeducation intervention called “Featback” [22], modeled on psychoeducation principles and including elements such as freedom to choose modules, automated messages, and feedback on monitoring and reflective exercises. The authors evaluated effectiveness and experiences of this intervention in a sample of 354 female participants (mean age = 24.2 years) with any ED in four conditions: Featback alone, Featback with low intensity therapist email support, Featback with high intensity therapist support, and waitlist control. They found that Featback with or without added support was superior to waitlist for bulimic symptoms (*d* = 0.16) and for ED-related quality of life at 3-month follow-up (*d* = 0.22) but was similar to waitlist for symptoms of AN. Although additional therapist support did not have an impact on treatment effectiveness, it did significantly increase participants' level of satisfaction with the program [22].

The remaining two articles examined diverse types of online self-help programs for young people with EDs. Wilksch et al. [71] conducted an RCT comparing Media Smart-Targeted, an online program that addresses known ED risk factors, to receiving a one-off email with tips for fostering a positive body image for participants with any ED (*n* = 316, age range = 18–25 years). The researchers found that participants who received Media Smart-Targeted were 75% less likely than those who received tips for body image to meet diagnostic criteria for an ED 12 months after the intervention [71]. In a non-randomized study in participants with AN, the authors [30] examined the effectiveness of treatment as usual when supplemented by videos on ED recovery (i.e., self-help component), daily text messages, and one weekly in-person meeting (n = 22, mean age = 25 years). Results showed significant reductions in participants’ global (∆η2 = 0.59, corresponding to a large effect size) and subscale (∆η2 range = 0.41–0.60, corresponding to large effect sizes) scores on the EDE-Q [30].

#### Registered protocols

The review identified several recently registered protocols outlining studies that fit our inclusion criteria but did not yet have published results. There were two protocols outlining RCTs to evaluate the smartphone apps, *Recovery Record* [65] and *Jourvie Research app* [62, 63] for participants with AN. A third study proposed an RCT evaluating a CBT-based self-help program, with and without therapist support, for participants with BN [57]. A fourth RCT proposed to evaluate the fully automated, online psychoeducation program *Featback*, comparing groups with and without expert patient support [64]. A fifth study proposed an uncontrolled trial to evaluate the transferability of FBT for adolescents with AN to a videoconference platform [60].

The review also identified several registered protocols with RCT methodologies evaluating self-help interventions, that met inclusion criteria despite published results no longer meeting criteria, for example, in the case that protocols targeted individuals potentially in the age range of interest, but eventual recruitment led to a mean participant age greater than 25 years [58, 59, 61, 66]. Nonetheless, similarly rigorous methodologies evaluating self-help in youth would contribute to evidence on this type of intervention.

### Modes of delivery: qualitative and mixed-methods studies:

The qualitative and mixed-methods studies described below give insight into participant perspectives on the use of virtual modalities of care and on the transition to virtual treatment during the COVID-19 pandemic.

#### Mobile phone applications

Four studies explored participant perspectives on the use of four different smartphone applications, *Recovery Record* [46, 50], *TCApp* [48], and a recently developed unnamed app [51]. Two studies describing the development of new smartphone applications for EDs were also identified (no data was collected) [40, 72].

During initial application development for *Recovery Record,* acceptability data was collected in a naturalistic manner through a survey that was available to any individual who downloaded *Recovery Record* from the app store. Over a 2-year period, 97% of those who rated the app (*n* = 2503) gave it at least a 4/5 rating [46]. Using a naturalistic and cross-sectional study, Lindgreen et al. [50] described the experiences of participants with either AN or BN (*n* = 41, mean age = 24 years) using the Recovery Record self-monitoring app in addition to treatment as usual, which was either weekly family therapy or group/individual sessions. Rating of three key app features, namely meal logs, phone notifications (i.e., meal reminders and positive affirmations), and data sharing with clinicians varied across participants and ranged from supportive to obstructive of daily life. Furthermore, the authors found that participants' experiences were mixed and depended on several variables including their appreciation of app features, the frequency of app usage, and the context in which the app was used (e.g., home *vs*. school).

Anastasiadou et al. [48] used group discussions with stakeholders, including participants (*n* = 9, mean age = 15 years) who were drawn from an RCT evaluating the effectiveness of the TCApp smartphone app plus treatment as usual *vs*. treatment as usual alone in participants with any type of ED, over a 12-week period (National Library of Medicine, NCT03197519). All participants reported that they found the app practical and easy to use, despite some problems with the design of the app, particularly the over-quantification of symptoms and the lack of personalization. Six of nine participants rated the content appropriate, explaining that the app helped gain better understanding of problematic behavior and was a good companion during recovery. There were limited privacy and anonymity concerns (three out of nine) and some (three out of nine) found the contact with therapists in the app limited and impersonalized.

Preliminary acceptability of *Space from Body and Eating Concerns Program,* a mobile phone and desktop application using CBT principles for the treatment of any ED in adolescents (*n* = 44, age = 14–17 years), was described [41]. Overall, more than half of participants provided positive feedback relating to perceptions that the app would have a positive impact on ED recovery and would be useful as compared with in-person treatment. Negative or constructive feedback included suggestions for improving app content and design and adding gamification features.

Finally, one study examined user experience of a new smartphone app for adolescents with AN [51]. The app has an unguided self-help program based on psychoeducation, CBT, and motivational interviewing. In their study, the authors recruited 8 female adolescents between 12- and 18-years-old (mean age = 15.5 years) as well as clinicians to participate in semi-structured focus groups. Qualitative data showed that the app was viewed positively by both patients and clinicians, although concerns were raised surrounding novelty, dependability, and efficiency. Overall, the psychoeducation portion was deemed important by both patients and clinicians [51].

#### Internet self-help

Participant views on an internet-based cognitive behavioral self-help treatment with email support from a therapist were described by Sanchez-Ortiz et al. [52]. Purposive sampling was used to select individuals with diverse fields of study in university, with a diagnosis of BN or Eating Disorder Not Otherwise Specified (EDNOS), and who had completed between four and eight sessions of the intervention from among a larger group of 76 women participating in an RCT testing the effectiveness of iCBT compared to a delayed treatment control for EDNOS or BN [52]. Nine participants took part in semi-structured interviews while 31 of the 64 RCT participants who completed at least one session responded to an online questionnaire. All participants mentioned that the treatment improved symptoms and eating patterns in some way. Treatment flexibility was considered important, participants felt supported in their treatment, and the internet-based self-help program was user-friendly and relevant. However, 68% of participants expressed lack of privacy as the biggest problem with being able to access treatment when they wanted to.

#### Transition to virtual treatment during the COVID-19 pandemic

Several articles describe the transition to virtual care during the pandemic [15, 16, 18, 39, 44, 45, 47, 49, 53]. Many ED clinics were able to transfer most of their services online [15, 16, 18, 39, 44, 45, 47, 49]. Parents seemed to be the most appreciative of having appointments virtually. On the other hand, patients and staff often preferred face-to-face compared to online contact [43, 49, 53] and most patients reported wanting to return to in-person appointments for most components of treatment when pandemic-related restrictions would be lifted [15, 44, 49, 53]. Shaw [43], for example, described the transition of an art psychotherapy program for adolescents with AN to videoconference format, overall, as feasible but hindering connectedness and communication, as occasionally facilitating participant disengagement, and as potentially causing difficulties for participants with AN who struggle with seeing themself and being seen on camera as it may perpetuate negative thoughts related to EDs. Nonetheless, in several cases, patients found virtual treatment to be a positive experience [45, 49, 53] and rated the impact of technology on their treatment experience as low [53]. There may also be additional advantages to online care, such as providing opportunities for exposure to triggers in the home environment and allowing patients on bed restrictions to participate in group therapy at a distance [15]. In addition, emerging observational data suggests that the transition from face-to-face to telehealth treatment can be associated with a decrease in ED symptoms, comparable to historic benchmarks for in-person treatment [45]. Shaw et al. [53] found that there were more referrals accepted and an increase in telemedicine appointments offered in 2020 compared to 2019.

#### Case studies

One case study (*n* = 2, ages 15 and 20 years) describing a multidisciplinary intervention including FBT for youth with AN delivered via videoconference and unlimited instant messaging support online found that the participants gained 1.9 and 2.9 kg and their ED symptoms decreased (7 and 12-point decrease on the EDE-Q) [54]. Another case study [73] describing the provision of CBT via videoconferencing for one 14-year-old female participant with EDNOS found that the intervention, which included one of twelve sessions in-person, was acceptable to the participant and her family and resulted in increased food intake and normalization of the participant’s growth trajectory.

#### Other technologies

Marco et al. [32] compared CBT with and without an adjunct virtual reality therapy component targeting body image disturbance in an RCT (*n* = 34 females, mean age = 21.82 years). The VR component consisted of an adaptation [74] of an in-person CBT for body image program [75] that combined group sessions and individual psychotherapy sessions which immerse participants in environments and exercises that are intended to help them become aware of and change their body image [32]. All participants, regardless of treatment group, experienced significant improvements in body image (*η*^2^ = 0.35, corresponding to a large effect size) and ED psychopathology (*η*^2^ = 0.70, corresponding to a large effect size) post-treatment and at 1-year follow-up, although participants receiving the VR intervention showed greater improvement. An RCT examined the effectiveness of having text messages as additional support to in-person CBT treatment [35]. Ten females (mean age = 21.5 years) who met diagnostic criteria for AN were exposed to both treatment conditions, either receiving or not receiving motivational text-messages in a randomized order, using a replicated single-case alternating treatment design. The authors found no significant main effect of additionally receiving text messages on caloric intake or dietary restraint [35].

## Discussion

### Main findings

This scoping review aimed to describe how technology is currently being included in interventions to treat youth with EDs, to categorize interventions in terms of hybrid versus fully online programs, and to evaluate the effectiveness of such interventions. We reviewed both quantitative and qualitative/mixed-methods/observational data in addition to published protocols. The number of studies for each specific treatment modality was limited, but the majority of quantitative and qualitative studies were considered of high quality based on our systematic quality assessments.

Overall, most TBIs described in the included studies were used as primary treatments, although supplementary support was also available in most studies. The modalities, especially videoconferencing, appeared to have been used to deliver evidence-based therapies (e.g., FBT). Additionally, TBIs were more often used to deliver therapy rather than medical or other health services. Finally, most of the interventions included were completely online, as opposed to hybrid interventions.

Our review identified few studies evaluating the use of videoconferencing to deliver psychotherapy for youth with EDs. This is a clear gap in the literature given the ease with which in-person therapy can be adapted for this medium and the rapid shift to this modality during the COVID-19 pandemic. Indeed, studies describing the transition to online services during the pandemic revealed that most ED outpatient programs and eventually day programs were able to move entirely online quickly and efficiently and that most patients and families see some important benefits with videoconferencing [15, 16, 18, 39, 44, 49, 53]. Though shown to be acceptable, studies on patient experience of virtual care provide nuance on how the accessibility and flexibility of virtual care must be balanced with the decreased connection patients and clinicians may report experiencing in exclusively online treatment. Some studies pointed to the necessity of face-to-face meetings early in treatment to build rapport while others, specifically referencing the COVID-19 period, prioritized those with the highest psychological needs for in-person care. Still, these services may be useful to other groups including those with reduced mobility or living in more remote areas and can be used according to the desires of individual patients, families, and clinician teams, within a hybrid framework that may become an essential part of the post-pandemic landscape.

It should be noted that in all three of the empirical studies on videoconferencing, the modality was new, but the specific type of therapy used already has a good evidence base when delivered in-person. Thus, FBT, a well-established treatment for adolescents with AN, may be effective for weight gain (in those who are underweight) and for decreasing ED symptoms in adolescents with AN when delivered virtually. However, these findings are drawn from only one case study and two quantitative studies, neither of which use in-person therapy as a control group comparison. Interestingly, available data of one study suggest that FBT delivered virtually may be as effective as online guided self-help FBT for decreasing ED symptoms [31]. This could point to the transferability of FBT to various modalities, which could increase treatment options for patients and improve accessibility, though these findings are preliminary. Though more rigorous studies are needed to fully substantiate the effectiveness of videoconferencing therapy for youth with EDs, especially in other populations than youth with AN, these initial findings are in line with studies that show videoconference therapy to be effective for other mental disorders, including depression and obsessive–compulsive disorders [76, 77].

The limited number of studies identified in this review evaluating mobile applications as treatment modalities for youth with EDs did not find statistically significant effects of this intervention on eating-disorder related outcomes. This is in agreement with literature on the topic which found that while mobile apps are generally acceptable to patients, they have limited effects on ED psychopathology [78]. This could represent a true lack of effect or could represent an inability to detect the specific contributions of the app to recovery given that patients in both interventions discussed here continued to receive therapy as usual in addition to using the app. The small number of studies evaluating mobile applications in youth is also somewhat surprising considering the many different mobile applications available for ED care [79, 80] and considering that youth, especially females, are amongst the biggest users of mobile phones and applications [81]. Given this context, future research may consider the impacts of unexamined use of mobile apps for EDs, especially because mobile apps which track calories have been associated with increased ED symptomatology [82–84].

Clinicians and patients agree that mobile apps are useful in early stages of the disease [51] and features such as psychoeducation and self-monitoring can be relevant at these points. The ease with which they can be implemented [80] also means that there is high potential to providing these interventions early in the course of treatment. Patient experiences with mobile apps suggest that the option to personalize features in the app is critical and emerging research suggests that multimedia features and the possibility to easily link to resources are the most engaging to youth patients [51, 85]. Beyond this, the use of apps and their specific features should be discussed on a case-by-case basis that takes into account patient and clinician preferences and capacities, as these can vary greatly between individuals [50].

While there has been no systematic evaluation of self-help interventions specifically for adolescents and young adults, early reviews that included mixed age populations [10–13] found that these interventions were superior to wait-list for reducing ED psychopathology, especially for participants with binge-type symptoms (vs. restrictive-type symptoms). However, these reviews recommended that more RCTs should be carried out to confirm the effectiveness of such interventions, especially in adolescents. More recently, Ahmadiankalati et al. [13] maintained that evidence for both acceptability and effectiveness of internet-based interventions was limited and that there is a lack of studies in adolescents, among other groups.

Similarly, we found that all self-help interventions were targeted towards older adolescents (> 16 years) and young adults. The pertinence, effectiveness and safety of this modality for younger adolescents, thus, remains unknown. Evidence for the use of self-help for patients with AN is also limited, based on our study though there may be some benefits in terms of weight gain [27, 28, 31]. As for restrictive ED symptoms, findings were mixed, ranging from a decrease to no change or even an increase in restrictive symptoms from baseline to end of treatment [22, 28, 30, 71]. It is noteworthy to mention the studies in which symptoms decreased were either uncontrolled or used a passive placebo group.

Effectiveness of internet self-help, and internet CBT in particular, for patients with BN and other purgative type EDs is promising but still needs to be verified as compared to active control interventions. The one study that compared internet self-help to an active control found that [34, 35] internet CBT was effective but had similar outcomes as the control intervention. When compared to waitlist control, we identified studies that confirmed effectiveness, but this was true, with large effect sizes and clinical improvements, in under half of study participants.

Finally, it should be noted that many of the studies examining internet self-help intervention were published between 2011 and 2013, which may lead us to question the current pertinence of this modality for individuals with EDs. Despite this, we did identify two protocols for internet self-help published in 2020 and 2021. Further, a smaller number of self-help studies, published over the last 6 years, were identified. Thus, this modality appears still relevant but has most likely been overshadowed by the development of new mobile apps and the increase of videoconferencing use since the beginning of the pandemic. Nevertheless, it may be an important option for those who would not seek in-person care. With regards to internet self-help of all types, design may be an important feature and interventions incorporating multimedia elements, that is, a combination of audio, text, video, etc., are associated with the greatest ED symptom reduction in treatment groups [85]. Though technology design was not a key element of our research, interventions explicitly described as interactive and/or multimedia in manuscripts were effective [22, 30, 71]. Even so, results should be interpreted cautiously as the improvements seen in the study by Kim [30] cannot be attributed to the multimedia intervention alone, as patients continued to receive TAU and weekly in-person guidance for technology usage.

The five protocols with unpublished results identified in this review target various modalities of intervention including two protocols on mobile applications, two on internet-based self-help programs, and one on videoconference therapy, with the majority (4/5) using rigorous RCT methodology. Future studies may build on the methodologies and interventions described. Additionally, the four published protocols with results not meeting our review inclusion criteria are informative and may be of interest to researchers and clinicians in that all use RCT methodology and most use active control groups, which can be applied to the population of interest.

Overall, the current findings align with previous reviews [10–13] that have found promising results for TBIs. Similar to what was described in these reviews, there seems to be few studies evaluating mobile application effectiveness while videoconferencing and internet self-help have been evaluated in a variety of contexts. Our review adds to previous work in that we reported on TBI use during the COVID-19 pandemic, showed the lack of studies on adolescents and youth, and brought to light the lack of hybrid intervention models.

### Strengths and limitations

This scoping review has several strengths. First, the review has a rigorous methodological procedure, including a quality assessment for both quantitative and qualitative studies to provide key information on the studies included. Second, contrary to a previous review of TBIs [11], our age criteria allowed us to review the literature in youth and emerging adults in the most extensive way without including studies that are not pertinent. Nevertheless, we had to compromise on age specificity in some cases where the age range extended beyond 25 years. There was a need for such information as this population is particularly at risk for EDs and has distinct developmental characteristics. Further, the present review included literature from before and during the pandemic and studies were categorized in terms of modes of delivery as well as described in terms of being hybrid versus fully online interventions.

Several limitations need to be considered. First, no cross-coding was carried out during the title and abstract screening phase, which may have had an impact on initial article selection for the study. However, we used a conservative approach to screening, which should have mitigated this impact. Second, the inclusion criteria were relatively strict in terms of age and diagnosis, which led to exclusion of studies that could potentially be relevant for researchers and clinicians interested in subclinical and/or older adult populations. However, our use of an inclusion criteria based on average participant age (between 10 and 25 years) also means that studies including individuals outside this range may also be included. Third, only results from the last 10 years were included based on the rationale that older studies may not have up to date technologies. It is possible that some studies prior to 2011 had similar methodologies and interventions to the ones we included, however, these were likely included in earlier reviews of virtual interventions, such as Aardoom et al. [10]. Fourth, several included studies had a relatively small sample size and lacked a control group, which could make the generalization of findings difficult. Fifth, evaluating dropout rates, which are highly variable (4.7–84.8%) in studies evaluating TBIs for EDs [11], warrants further investigation. Finally, as the number of studies for each treatment modality was relatively small and individual studies were highly heterogeneous, we did not further quantify overall results by using meta-analytic procedures. Such an approach would be useful once more empirical studies become available.

This review identified several areas for future research. Studies are needed on videoconference therapy to better understand if common in-person treatment approaches, such as FBT and CBT, can be reasonably transferred online without compromising effectiveness. This is necessary considering this modality was used most frequently when transitioning to online treatment during the COVID-19 pandemic. In our review, mobile phone applications were not associated with improved treatment outcomes in youth when added to treatment as usual. The limited number of studies in clinical populations overall makes it difficult to draw clear conclusions on the pertinence of this modality for treatment. It is possible that the features provided in current mobile phone apps (mostly self-monitoring and food logs) may not be of benefit in a clinical youth population and that other features may need to be proposed. There was also a concern regarding the lack of individualization of available apps, which may make their use less appealing to patients. Self-help was useful in all cases but its effectiveness in comparison to active treatment modalities remains unclear, and more studies need to be conducted to determine the level of support (high vs. low intensity) that is required for effectiveness and the type of support that is the most helpful (automated *vs*. clinician *vs.* technician). Additionally, there is a lack of systematic studies on hybrid treatments, which warrants further investigation considering patients’ expressed needs/desires for in-person support when receiving virtual care. The combination of in-person and virtual components of treatment may offer patients the opportunity to benefit from the best elements of each approach. However, evidence-based guidelines on providing hybrid care are lacking [86] and no firm conclusions can be made based on our review as most of the studies reviewed were fully online.

## Conclusions

This review has found evidence supporting the use of TBIs for the treatment of EDs in youth. In particular, internet self-help guides were useful for decreasing ED symptomatology and the evidence to support this finding was of high quality. Though qualitative studies suggest that some form of therapist support is an essential element of treatment, future studies could investigate the impact of therapist support on ED outcomes in self-help programs. In addition, more controlled trials with larger samples are required to evaluate the effectiveness of videoconference therapy. However, videoconferencing has been shown to be feasible and acceptable during the COVID-19 pandemic. Limited evidence was found on the use of mobile phone applications in the clinical youth population. More controlled trials of mobile phone applications in clinical populations are required to conclude on their effectiveness. Considering that youth are particularly at risk for EDs, there is a lack of high-quality RCTs testing virtual care and hybrid interventions in this population. Filling this important research gap could lead to significant improvements in ED care in a rapidly evolving landscape of ED treatment in youth.

## Supplementary Information

**Additional file 1: Fig. S1.** Quality assessment of quantitative studies.

**Additional file 2: Fig. S2.** Quality assessment of qualitative studies.

## Data Availability

As no new data were generated in this study, data sharing is not applicable to this article.

## Abbreviations

AN: Anorexia nervosa
BMI: Body mass index
BN: Bulimia nervosa
CBT: Cognitive behavioral therapy
DSM-IV: Diagnostic and statistical manual of mental disorders fourth edition
DSM-5: Diagnostic and statistical manual of mental disorders fifth edition
ED: Eating disorder
EDE: Eating disorder examination
EDE-Q: Eating disorder examination-questionnaire
EDNOS: Eating disorder not otherwise specified
FBT: Family-based treatment
RCT: Randomized controlled trial
TAU: Treatment as usual
TBI: Technology-based interventions
