# Supplementary material for: The use of technology in the treatment of youth with eating disorders: A scoping review
Source: J Eat Disord. 2022 Nov 24;10:182. doi: 10.1186/s40337-022-00697-5 (PMC9700893; doi:10.1186/s40337-022-00697-5)
Supplement: Supplementary file 1 — Additional file 1: Fig. S1. Quality assessment of quantitative studies. [file 40337_2022_697_MOESM1_ESM.pdf]

Supplementary figure 1: Quality assessment of quantitative studies

	Aardoom et al. 2017	Anastasiadou et al. 2019	Anderson et al. 2017	Carrard et al. 2011	Fichter et al. 2012	Giel et al. 2015	Kim et al., 2019	Marco et al. 2013	Neumayr et al., 2019	Sanchez-Ortiz et al., 2011	Shingleton et al., 2016	Tregarthen et al., 2015	Wagner et al. 2013	Wagner et al., 2013 (2)	Wagner et al., 2014	Wilksch et al., 2017	Lock et al., 2021	Fichter et al., 2013
**1.** Were eligibility criteria for study entry prespecified and clearly described?																		
**2.** Was there absence of co-intervention that may influence outcome?																		
**3.** Were all eligible participants that met the prespecified entry criteria enrolled?																		
**4.** Was the intervention clearly described and delivered consistently across the study population?																		
**5.** Were 80% of people randomized included in the final analysis?																		
**6.** Was information on people who withdrew given?																		
**7.** Was an ITT analysis included?																		
**8.** Is there absence of evidence that more outcomes were measured than reported?																		
**9.** Were the outcome measures clearly defined, valid, reliable, and assessed consistently across all participants?																		
**10.** Did they discuss power or include effect size?																		
**11.** Do the authors control for confounders (either statistically, through matching or randomization)?																		
**12.** Did the statistical methods examine changes in outcome measures from before to after the intervention (change, not difference score)?																		
**13.** Were p-values for the pre to post change reported for all outcomes?																		
**14.** Were outcome measures of interest taken multiple times before the intervention and multiple times after (time-series design)?																		
**15.** Was the method used to assign participants to group truly random?																		
**16.** Was the number of participants randomized stated?																		
**17.** Were participants blinded to treatment allocation?																		
**18.** Were outcome assessors blinded to treatment allocation?																		
**19.** Was the success of the blinding procedure assessed?																		
**TOTAL OF YES & PARTIALLY (/19)**	12	14	8	12	16	7	10	14	12	15	10	3	13	10	9	11	15	13

LEGEND: green = yes, red = no, yellow = partially, gray = not applicable, white = not reported
