# Supplementary material for: The use of technology in the treatment of youth with eating disorders: A scoping review
Source: J Eat Disord. 2022 Nov 24;10:182. doi: 10.1186/s40337-022-00697-5 (PMC9700893; doi:10.1186/s40337-022-00697-5)
Supplement: Supplementary file 2 — Additional file 2: Fig. S2. Quality assessment of qualitative studies. [file 40337_2022_697_MOESM2_ESM.pdf]

Supplementary Figure 2: Quality assessment of qualitative studies

	Anastasiadou 2019	Kasson 2021	Naccache 2021	Sanchez-Ortiz 2011	Brothwood 2021	Stewart 2021	Shaw 2021	Lindgreen 2018
1. Was there a clear statement of the aims of the research?	green	green	green	green	green	green	green	green
2. Is a qualitative methodology appropriate?	green	green	green	green	green	green	green	green
3. Was the research design appropriate to address the aims of the research?	green	green	green	green	green	green	green	green
4. Was the recruitment strategy appropriate to the aims of the research?	green	green	green	green	green	green	green	green
5. Was the data collected in a way that addressed the research issue?	green	yellow	green	green	green	green	green	green
6. Has the relationship between researcher and participants been adequately considered?	yellow	yellow	yellow	yellow	green	yellow	yellow	yellow
7. Have ethical issues been taken into consideration?	green	yellow	green	green	green	green	green	green
8. Was the data analysis sufficiently rigorous?	green	yellow	green	green	green	green	yellow	green
9. Is there a clear statement of findings?	green	green	green	green	green	green	green	green
10. How valuable is the research?	green	green	green	green	green	green	green	green
TOTAL OF CRITERIA MET (/10)	9	6	9	9	10	9	8	9

LEGEND: green = yes, yellow = can't tell, red = no
